# Thyroid Storm With Multiorgan Dysfunction Mimicking Sepsis: Thionamide-Sparing Bridge to Delayed Carbimazole

**DOI:** 10.7759/cureus.103391

**Published:** 2026-02-10

**Authors:** Sunita Kumari, Balwant Kumar, Bodhisatwa Choudhuri, Anindya Dasgupta, Manisha Modi, Arnab Dey, Rajan Palui, Amarta Shankar Chowdhury, Parul Agrawal

**Affiliations:** 1 Accident and Emergency Medicine, The Mission Hospital, Durgapur, IND; 2 Medicine, The Mission Hospital, Durgapur, IND; 3 Critical Care, Emergency Medicine, and Rheumatology, Parkview Super Specialty Hospital, Kolkata, IND; 4 Critical Care Medicine, The University of Edinburgh, Edinburgh, GBR; 5 Emergency Medicine, Narayana Hospital (NH) Barasat, Barasat, IND; 6 Critical Care Medicine, The Mission Hospital, Durgapur, IND; 7 Gastroenterology, The Mission Hospital, Durgapur, IND; 8 Endocrinology and Diabetes, The Mission Hospital, Durgapur, IND

**Keywords:** acute liver injury, atrial fibrillation, cholestyramine, graves’ disease, hyperthyroidism, lithium, multiorgan dysfunction, procalcitonin, thionamide-sparing therapy, thyroid storm

## Abstract

Thyroid storm represents a critical exacerbation of thyrotoxicosis, often associated with Graves' disease, characterized by fever, gastrointestinal disturbances, neuropsychiatric manifestations, and tachyarrhythmias, closely resembling sepsis in its clinical presentation. The elevation of inflammatory biomarkers can be significant, frequently leading to delayed or missed diagnosis. Conventional treatment (beta-blockade, thionamides, iodine after thionamide, and corticosteroids) involves rapid management of adrenergic symptoms alongside inhibition of thyroid hormone synthesis and release; however, severe hepatocellular injury and coagulopathy may limit early thionamide administration, necessitating alternative "bridging" therapeutic strategies. We present the case of a 31-year-old male patient with no prior history of thyroid disease who presented with fever and vomiting for three to four days, followed by agitation and palpitations, subsequently developing atrial fibrillation with rapid ventricular response (150 beats per minute (bpm)). Laboratory investigations revealed significant hypokalemia (1.9 mmol/L), markedly elevated procalcitonin (58.1 ng/mL), and severe hepatocellular injury with coagulopathy, as evidenced by elevated aspartate aminotransferase (AST; 3740 U/L), alanine aminotransferase (ALT; 1232 U/L), and international normalized ratio (INR; 2.06). Thyroid function tests demonstrated suppressed thyroid-stimulating hormone (TSH; <0.01 mIU/L), elevated free thyroxine (free T4; >90 pmol/L), and positive thyroid-stimulating hormone receptor antibodies (TSH-receptor antibodies; 19.5), supporting a diagnosis of Graves’ disease, while ultrasound and scintigraphy were consistent with the diagnosis. The diagnosis of thyroid storm was established using the Burch-Wartofsky Point Scale (BWPS; 70) and Japanese Thyroid Association (JTA) criteria. Given the hepatic dysfunction, initial management deferred thionamide therapy; treatment comprised propranolol, corticosteroids, cholestyramine, and lithium, along with supportive intensive care and empirical antibiotics. Carbimazole was initiated on day 8 following hepatic improvement, and the patient was discharged on day 12, demonstrating normal liver function and thyroid hormone levels at two-week follow-up. This case illustrates that thyroid storm can closely mimic sepsis with markedly elevated inflammatory biomarkers and may be complicated by severe hepatocellular injury. A thionamide-sparing approach, when thionamides are contraindicated (e.g., agranulocytosis, hypersensitivity reactions, or severe hepatic dysfunction), utilizing lithium and cholestyramine can effectively serve as a bridging strategy, facilitating postponement of thionamide therapy when immediate antithyroid treatment is contraindicated.

## Introduction

Thyroid storm represents the most severe form of thyrotoxicosis, characterized by acute, life-threatening decompensation with systemic hypermetabolism and multi-organ dysfunction. Its clinical and laboratory phenotype can overlap substantially with sepsis (fever, tachycardia, hypotension/shock, and raised inflammatory markers), increasing the risk of diagnostic anchoring and delayed targeted therapy. Despite advances in critical care and earlier recognition, it maintains significant in-hospital mortality and resource utilization, particularly when presentation is delayed or when precipitating factors are not managed promptly [1-3]. Graves' disease is the most common cause, with typical precipitants including infection, surgery or trauma, iodinated contrast exposure, withdrawal or non-adherence to antithyroid medications, and acute cardiovascular events [1,2].

Thyroid storm is primarily a clinical diagnosis, as no single biochemical marker can differentiate it from severe thyrotoxicosis. To facilitate standardized recognition, clinicians use structured tools like the Burch-Wartofsky Point Scale (BWPS) and Japan Thyroid Association (JTA) criteria [2,4]. These tools focus on manifestations in thermoregulatory, cardiovascular, gastrointestinal-hepatic, and central nervous systems. Early diagnosis is crucial, as clinical deterioration can occur rapidly and may be disproportionate to initial thyroid hormone levels, including progression to malignant tachyarrhythmias and severe neurologic dysfunction (seizures, coma).

Standard therapeutic approaches aim to (i) mitigate adrenergic overactivity, (ii) inhibit thyroid hormone synthesis and release, and (iii) decrease peripheral T4 to T3 conversion, alongside supportive care and addressing the underlying cause [1,2]. Thionamides are central to this regimen; however, management can be complicated by hepatic dysfunction, which may arise from severe thyrotoxicosis, low-flow states, or concerns about drug-associated hepatotoxicity. These circumstances require bridging strategies [1]. Adjunctive treatments, such as cholestyramine, which disrupts thyroid hormone enterohepatic recirculation, and lithium, which inhibits thyroid hormone release from the thyroid gland, have shown efficacy in accelerating biochemical improvement when used with conventional therapy. This approach may be beneficial when thionamides are delayed or contraindicated [5-7].

We present a case of thyroid storm associated with Graves' disease, complicated by severe hepatic biochemical abnormalities and malignant tachyarrhythmia requiring urgent cardioversion. Management involved a physiology-guided, stepwise approach incorporating early adjunctive measures and precise timing for antithyroid medication [1-7].

## Case presentation

A 31-year-old male patient with no known past thyroid disease presented to the emergency department with acute-onset palpitations, chest discomfort, dyspnea, recurrent emesis, and agitation. His medical history included mild intermittent bronchial asthma, treated with a budesonide-formoterol metered-dose inhaler (200/6 mcg per actuation). There was no history of chronic liver disease, paracetamol/acetaminophen exposure, or documented hypotension or shock at the referring facility. He was a non-smoker and did not consume alcohol; there was no known drug allergy and no family history of thyroid or autoimmune disease.

On examination, he was febrile, markedly tachycardic, and clinically dehydrated, with no peripheral signs or symptoms of heart failure. The patient was restless and agitated but without focal neurological deficits. Thyroid examination revealed no abnormalities, with no palpable goiter or thyroid tenderness and no thyroid eye disease indications. Overall, the early presentation had a sepsis-like hyperadrenergic phenotype (fever, marked tachycardia, agitation, and gastrointestinal symptoms), prompting parallel evaluation for infection and thyroid storm.

Cardiac evaluation

**Figure 1:**
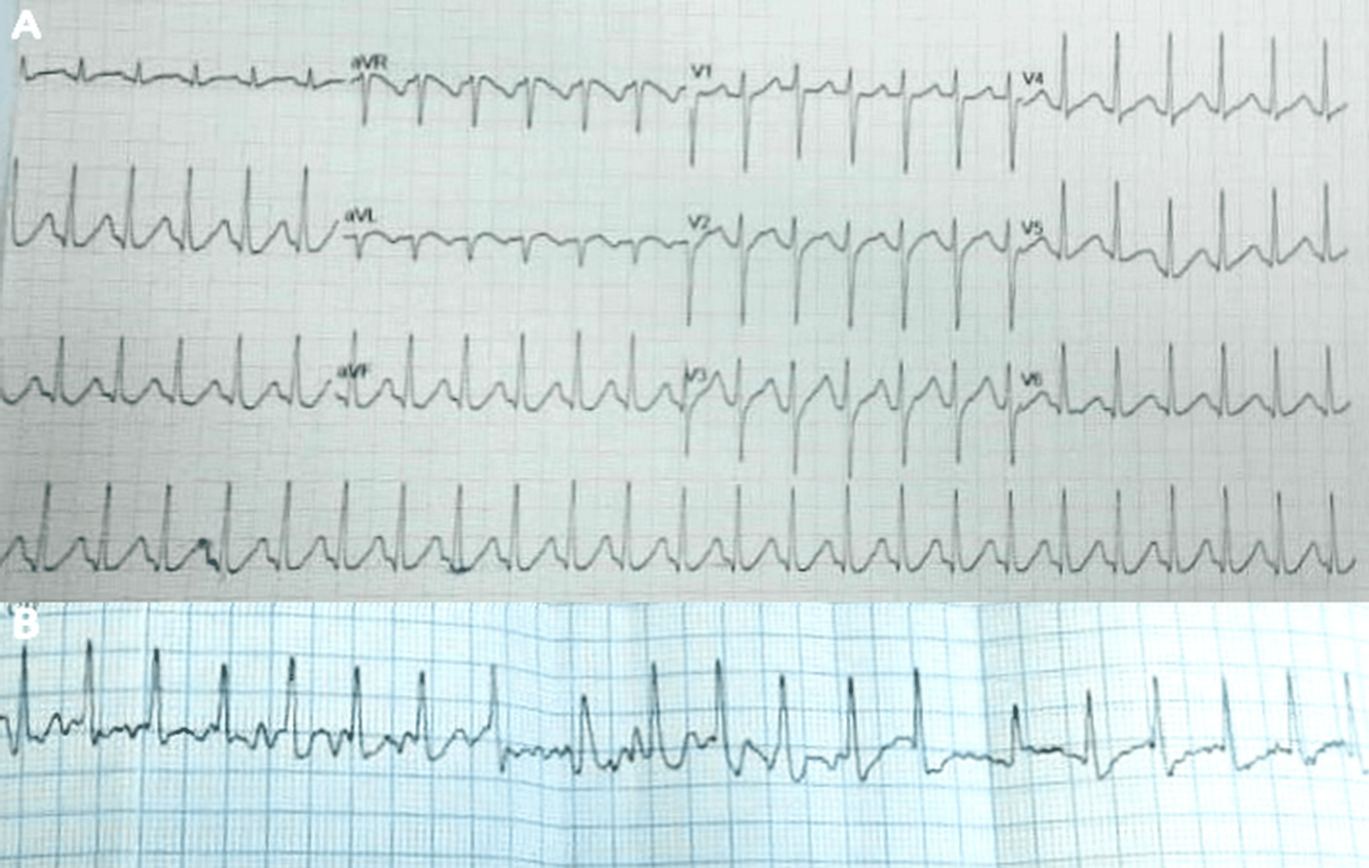
Electrocardiographic evidence of thyrotoxicosis-associated atrial tachyarrhythmia. (A) A 12-lead electrocardiogram during admission showing a regular narrow-complex tachycardia (~150 beats per minute (bpm)), consistent with 2:1 atrioventricular conduction. (B) Subsequent rhythm strip demonstrating an irregular narrow-complex tachyarrhythmia with variable R–R intervals, consistent with atrial fibrillation with rapid ventricular response.

On arrival at the emergency department, cardiac assessment was performed as part of initial stabilization. The initial ECG revealed supraventricular tachyarrhythmia, indicating atrial tachycardia with 2:1 AV conduction, which subsequently progressed to atrial fibrillation with rapid ventricular response (Figures 1A-1B). Due to persistent tachyarrhythmia, synchronized direct-current cardioversion was administered, restoring sinus rhythm after energy escalation. Bedside echocardiography showed hyperdynamic circulation without regional wall motion abnormalities or significant valvular pathology. Assessment of the inferior vena cava suggested intravascular volume depletion.

Investigations, diagnosis, and precipitant evaluation

Arterial blood gas analysis showed near-normal pH with mild metabolic acidosis (pH 7.377, PaO₂ 194 mmHg, PaCO₂ 38 mmHg, bicarbonate (HCO₃⁻) 21.3 mmol/L) and normal lactate (1.9 mmol/L). Initial biochemical assessments showed severe electrolyte imbalances, including profound hypokalemia, hypernatremia, and hypercalcemia, requiring urgent correction and monitoring. From admission, hepatic biochemical disturbance was evident, with marked transaminitis, evolving coagulopathy, and thrombocytopenia. Inflammatory biomarkers were elevated, particularly procalcitonin and C-reactive protein (CRP), which influenced empirical antibiotics despite no clear infectious focus. The progression of vital parameters and laboratory abnormalities is summarized in Table 1. These early trends, marked by transaminitis with coagulopathy and thrombocytopenia, alongside elevated inflammatory biomarkers, became key determinants of treatment sequencing, including initial deferral of thionamides.

**Table 1:** Serial trends in vital signs and key laboratory parameters during hospitalization and follow-up All abnormal values are shown in bold. Abbreviations: ALB, albumin; ALP, alkaline phosphatase; ALT, alanine aminotransferase; Anti-TPO, anti-thyroid peroxidase antibody; AST, aspartate aminotransferase; bpm: beats per minute; CPK, creatine phosphokinase; CRP, C-reactive protein; DBP, diastolic blood pressure; FT3, free triiodothyronine; FT4, free thyroxine; FU, follow-up; GGT, gamma-glutamyl transferase; Hb, hemoglobin; HR, heart rate; INR, international normalized ratio; K, potassium; N/A, not available/not tested; Na, sodium; PCT, procalcitonin; SBP, systolic blood pressure; Temp, temperature; TgAb, anti-thyroglobulin antibody; TLC, total leukocyte count; TRAb, thyroid-stimulating hormone receptor antibody; TSH, thyroid-stimulating hormone.

Parameter (units)	Day 1	Day 2	Day 5	Day 7	Day 12	Two-week FU	Six-week FU	Reference range
Temp (°F)	99	98.6	98.5	98.6	98.6	98.7	98.4	97.7–99.5
HR (bpm)	150	112	98	94	77	82	74	60–100
SBP/DBP (mmHg)	140/60	124/76	130/82	122/80	130/82	132/84	124/80	(90–130)/(60–80)
Hb (g/dL)	14.4	13.8	11.9	9.8	12.6	13.1	13.7	13–17
TLC (cells/µL)	5500	16300	10100	9600	9100	6940	7800	4000–11000
Platelets (×10³/µL)	155	130	40	78	154	186	250	150–400
Na (mmol/L)	153	160	146	137	133	136	142	135–145
K (mmol/L)	1.9	4.2	3.3	3.7	4.3	4.7	4.1	3.5–5
Ca (mg/dL)	12.1	10.8	9	N/A	8.5	8.8	9.1	8.4–10.2
Creatinine (mg/dL)	0.9	1.4	1.23	1.05	0.8	0.74	0.9	0.6–1.3
Total Bilirubin (mg/dL)	1	1.2	1.3	1.2	1	0.7	0.8	0.2-1.3
Direct Bilirubin (mg/dL)	0.5	0.4	0.3	0.2	0.1	0.1	0.2	0-0.3
AST (U/L)	1009	3740	906	98	26	31	22	10-40
ALT (U/L)	457	1232	1062	290	63	52	27	8-50
GGT (U/L)	30	26	30	28	32	27	30	8-36
ALP (U/L)	78	64	59	60	92	101	104	44-150
ALB (g/dL)	3	2.8	2.5	2.9	3.3	3.5	4.1	3.5-5
INR	1.47	2.06	1.41	N/A	1.12	1.02	0.98	0.8-1.2
CRP (mg/L)	84.1	75.4	12.6	11.7	7.4	2.1	1.6	0–5
Procalcitonin (ng/mL)	58.1	N/A	3.29	N/A	0.54	N/A	N/A	<0.05
CPK (U/L)	321	277	N/A	N/A	182	N/A	N/A	55-170
TSH (mIU/L)	<0.01	N/A	N/A	N/A	0.07	0.3	2.1	0.47–4.68
FT4 (pmol/L)	>90	N/A	N/A	76	51.4	21.8	13.2	10-22
FT3 (pmol/L)	>90	N/A	N/A	N/A	N/A	7.2	4.7	3.1-6.8
TRAb (IU/L)	19.5	N/A	N/A	N/A	N/A	1.7	0.5	<1.75
Anti-TPO (IU/mL)	92	N/A	N/A	N/A	N/A	N/A	N/A	<35
TgAb (IU/mL)	12.8	N/A	N/A	N/A	N/A	N/A	N/A	<40

Thyroid function tests showed severe thyrotoxicosis, with suppressed thyroid-stimulating hormone (TSH) and elevated free triiodothyronine (FT3) and free thyroxine (FT4) beyond assay reporting limits. Autoimmune markers indicated Graves' disease, with strongly positive thyroid receptor antibody (TRAb), elevated anti-thyroid peroxidase antibody (anti-TPO), and negative anti-thyroglobulin antibody (TgAb).

**Figure 2:**
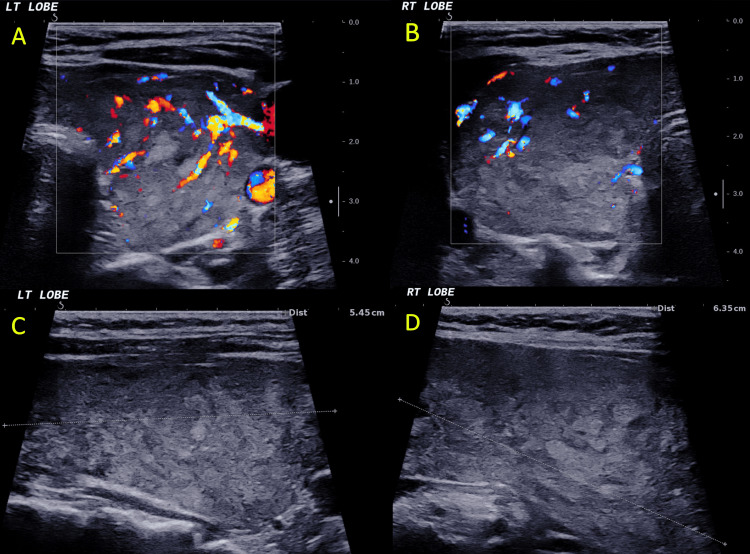
Thyroid ultrasonography with color Doppler demonstrating diffuse hypervascularity consistent with Graves’ disease. (A–B) Color Doppler images of the left (LT) and right (RT) thyroid lobes showing diffusely increased intraparenchymal vascularity (“thyroid inferno” pattern), supportive of Graves’ disease. (C–D) Corresponding grayscale (B-mode) longitudinal views demonstrating mild diffuse thyroid enlargement with a heterogeneous echotexture (longitudinal measurements: LT lobe ~5.45 cm, RT lobe ~6.35 cm).

**Figure 3:**
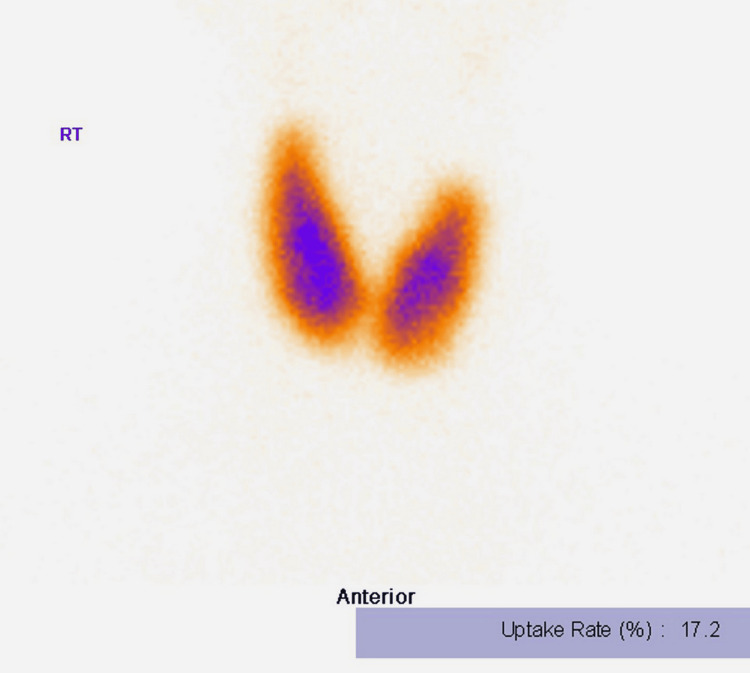
Thyroid scintigraphy demonstrating diffuse increased tracer uptake consistent with Graves’ disease. Anterior planar technetium-99m-pertechnetate thyroid scan showing diffusely increased, relatively homogeneous uptake in both thyroid lobes, without focal nodularity, supportive of Graves’ disease in the clinical context. The measured uptake rate was 17.2% (as displayed on the image). Abbreviation: RT, right

Neuroimaging was conducted due to agitation; computed tomography (CT) showed no acute intracranial pathology. Ultrasonography of the abdomen revealed no focal hepatic lesions, biliary dilatation, or obstructive pathology. Thyroid ultrasonography showed mild diffuse enlargement with heterogeneous echotexture. Color Doppler imaging demonstrated increased intrathyroidal vascularity in both lobes (Figure 2). Technetium-99m (^99m^Tc)-pertechnetate thyroid scintigraphy revealed diffuse, homogeneous increased tracer uptake with 17.2% uptake, consistent with Graves' disease (Figure 3).

A clinical diagnosis of thyroid storm was made based on structured criteria. The BWPS score indicated severe storm at 70 (Table 2), and the patient met JTA diagnostic criteria for thyroid storm (Table 3). Evaluation for common precipitants did not identify a definitive infectious source. Blood and urine cultures were negative, and the assessment did not localize sepsis. Viral hepatitis serologies (including hepatitis A and E IgM and hepatitis B and C screening) and autoimmune screening were negative, with no reported exposure to acetaminophen. Disseminated intravascular coagulation (DIC) was not supported by fibrinogen values or clinical bleeding, despite elevated D-dimer.

**Table 2:** Burch–Wartofsky Point Scale (BWPS) criteria BWPS scoring in this patient supports the diagnosis of thyroid storm [4]. Abbreviations:* *RVR, rapid ventricular response; bpm: beats per minute

BWPS domain	BWPS category (criteria)	Patient finding	Points
Thermoregulatory dysfunction	99.0–99.9 °F	99.0 °F	5
Central nervous system effects	Mild (agitation)	Agitation	10
Gastrointestinal/hepatic dysfunction	Moderate (nausea/vomiting)	Vomiting	10
Cardiovascular dysfunction (heart rate)	≥140 bpm	150 bpm	25
Atrial fibrillation	Present	Present (with RVR)	10
Congestive heart failure	Absent	Absent	0
Precipitating event	Present	Suspected infectious/sepsis-like trigger	10
Total BWPS score			70

**Table 3:** Japan Thyroid Association (JTA) diagnostic criteria JTA diagnostic criteria applied to this patient, confirming definite thyroid storm (TS1) [2]. Abbreviations: CNS, central nervous system; FT3, free triiodothyronine; FT4, free thyroxine; GI, gastrointestinal; TSH, thyroid-stimulating hormone; TS1, definite thyroid storm; bpm: beats per minute

JTA item	Criterion	Patient status
Essential	Thyrotoxicosis (elevated FT4 and/or FT3 with suppressed TSH)	Met (FT4 and FT3 above assay reporting limit; TSH suppressed)
Major feature 1	Central nervous system manifestation	Met (agitation)
Major feature 2	Fever ≥38°C	Not documented on admission (history of fever)
Major feature 3	Tachycardia ≥130 bpm	Met (150 bpm)
Major feature 4	Congestive heart failure	Not met
Major feature 5	Gastrointestinal/hepatic manifestation	Met (vomiting; marked hepatocellular injury/coagulopathy)
JTA category	TS1 (definite): Thyrotoxicosis + CNS manifestation + ≥1 major feature	TS1 (definite) met (CNS + tachycardia + GI/hepatic)

Differential diagnosis

Given the sepsis-mimicking phenotype with multiorgan dysfunction, several competing diagnoses were considered. Sepsis was felt less likely in the absence of a localizing focus and with persistently negative cultures, and antimicrobial therapy was guided by clinical reassessment. Acute hepatic injury differentials, including viral hepatitis and drug-induced liver injury, were evaluated and not supported by history or available investigations; ischaemic hepatitis was also considered, but there was no documented prolonged hypotension at the referring facility. Coagulopathy from severe DIC was considered because the D-dimer levels were high, but normal fibrinogen levels and no significant bleeding suggested it wasn't the cause. Overall, extreme thyrotoxicosis with a Graves’ autoimmune profile provided the most unifying explanation for the patient’s presentation.

Treatment course and outcome

The patient was admitted to the intensive care unit (ICU) and received supportive interventions, including temperature regulation and electrolyte correction. Hypokalemia was treated with IV potassium chloride infusion, with cardiac monitoring and electrolyte reassessment. Hypernatremia was corrected using 0.45% saline, monitoring the sodium correction rate. Hypercalcemia was managed with calcitonin 100 units subcutaneously thrice daily (TDS) and hydration. After cardiac rhythm stabilization, rate control was maintained with oral propranolol, titrated from 20 mg to 80 mg TDS, targeting a heart rate <100 beats/min. Therapeutic anticoagulation was withheld due to coagulopathy and thrombocytopenia, given the bleeding risk.

**Figure 4:**
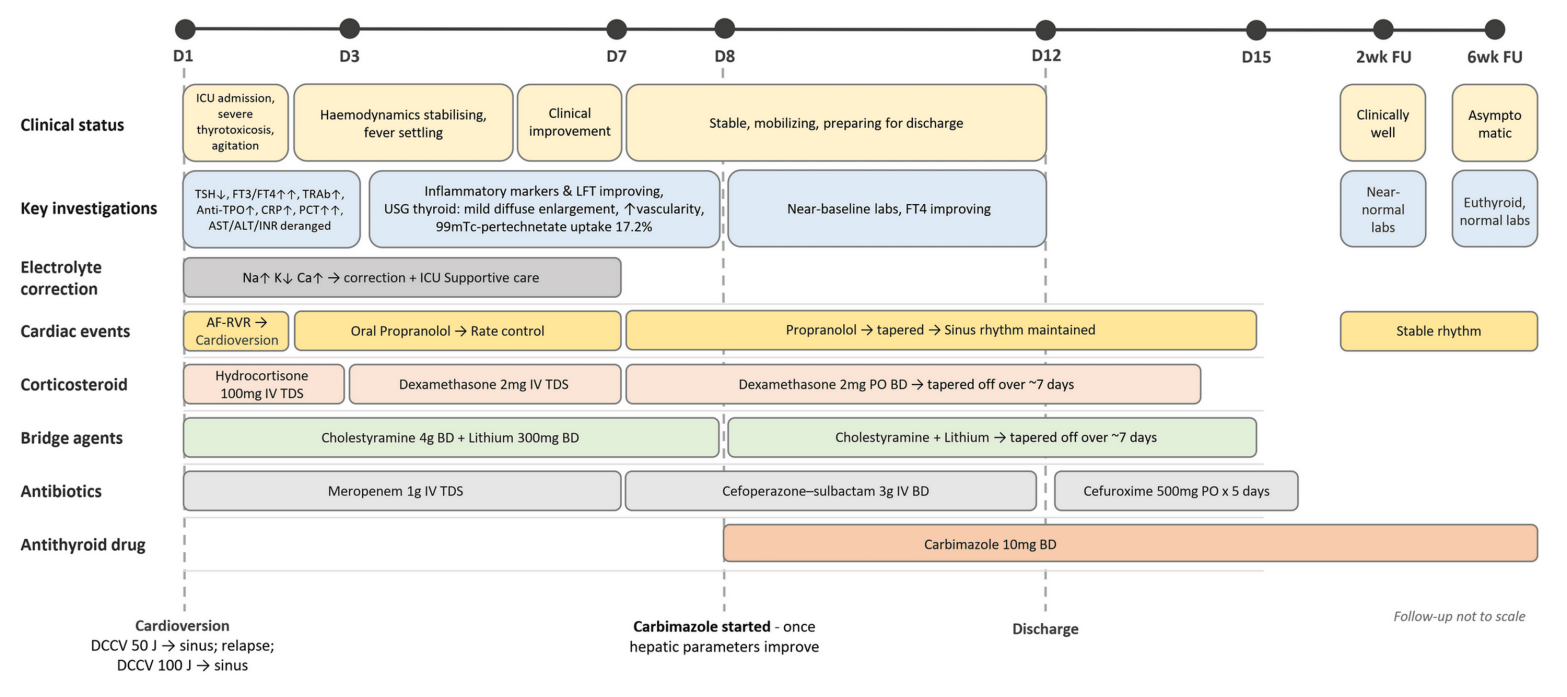
Timeline of clinical course, investigations, and treatment in thyroid storm. Timeline showing key symptoms, diagnostic investigations, major treatments, and follow-up milestones during hospitalization and subsequent review. Abbreviations: AF-RVR, atrial fibrillation with rapid ventricular response; ALT, alanine aminotransferase; Anti-TPO, anti–thyroid peroxidase antibody; AST, aspartate aminotransferase; BD, twice daily; Ca, calcium; D, day; DCCV, direct-current cardioversion; FT3, free triiodothyronine; FT4, free thyroxine; ICU, intensive care unit; INR, international normalized ratio; IV, intravenous; J, Joules; K, Potassium; LFT, liver function tests; Na, sodium; PCT, procalcitonin; PO, per os (oral/by mouth); TDS, three times daily; TRAb, thyrotropin receptor antibody; TSH, thyroid-stimulating hormone; USG, ultrasonography

A significant therapeutic challenge was presented by the hepatic biochemical derangement and coagulopathy at presentation, raising concerns about early thionamide initiation. A thionamide-sparing bridge was implemented. Hydrocortisone 100 mg IV TDS was given for three days, then replaced with dexamethasone 2 mg IV TDS after endocrinology consultation. From day 7, dexamethasone was changed to 2 mg orally twice daily (BD) and tapered over the following week. Cholestyramine 4 g BD and lithium 300 mg BD were started on day 1 as adjunctive therapies to reduce circulating thyroid hormones until thionamides could be safely introduced; both were tapered from day 8 (timeline summarized in Figure 4). Iodine therapy was not used due to the inability to reliably establish thionamide pretreatment during severe hepatic dysfunction; clinical stabilization was achieved with these measures.

Given critical illness and elevated inflammatory biomarkers (including high procalcitonin), empiric antimicrobial therapy was initiated with meropenem 2 g stat, followed by meropenem 1 g IV TDS. With negative cultures and no identifiable focus, antibiotics were de-escalated from day 7 to cefoperazone-sulbactam 3 g BD, and the patient was discharged on oral cefuroxime 500 mg BD for five days.

Outcome and follow-up

Clinical improvement was progressive, with stabilization of rhythm and resolution of neuropsychiatric and gastrointestinal manifestations. Hepatic and hematologic parameters showed improvement. Carbimazole 10 mg BD was started on day 8 as liver enzymes and coagulation indices improved, after which lithium and cholestyramine were tapered and stopped.

He was discharged on carbimazole and propranolol (10 mg TDS), with definitive therapy planned after recovery. At the two-week outpatient review, thyroid function, liver enzymes, coagulation profile, and platelet count improved and normalized by six weeks; follow-up values are summarized in Table 1.

## Discussion

Thyroid storm, though rare, is a high-mortality endocrine emergency diagnosed clinically, with biochemical severity as a supportive indicator. Guidelines emphasize early detection, immediate multimodal treatment, and investigation of triggers, as thyroid hormone levels may appear similar in uncomplicated thyrotoxicosis and thyroid storm [1-4,8]. Our patient presented with hyperpyrexia, atrial fibrillation with hemodynamic instability, gastrointestinal symptoms, and early encephalopathy with multiorgan dysfunction. The thyroid examination was normal with minimal signs of Graves' disease, highlighting that the absence of goiter or ophthalmopathy does not preclude Graves' storm in emergency contexts [1-4,8,9]. In our case, the lack of classic thyroid signs, combined with very high inflammatory biomarkers, initially reinforced a sepsis-first framing.

A significant complexity was severe hepatic dysfunction with coagulopathy, which limited therapeutic options. Liver test abnormalities are common in thyrotoxicosis and Graves' disease, ranging from mild transaminitis to cholestasis, congestive hepatopathy, ischemic injury, and rarely, acute liver failure, particularly in thyroid storm, where hypermetabolism and cardiocirculatory stress converge [10,11]. Mechanisms include hepatic hypoxia, venous congestion, thyroid hormone-mediated hepatocellular injury, and systemic inflammatory activation; severe hepatic injury can progress rapidly and may not correlate with free hormone levels [10,11]. In our patient, the combination of pronounced transaminitis, elevated INR, thrombocytopenia, and metabolic derangements indicated storm-associated organ dysfunction rather than drug-induced injury, given the absence of paracetamol exposure, negative viral/autoimmune hepatitis workup, and no hypotensive episodes at the referring facility. That liver phenotype became the practical constraint that forced us to reconsider the usual ‘thionamide-first’ sequencing.

**Figure 5:**
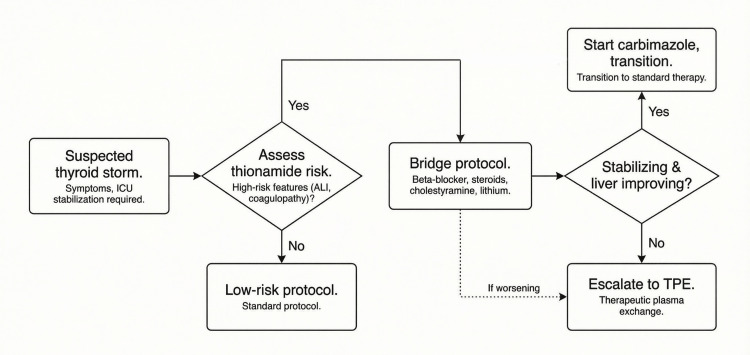
Algorithm for thionamide-sparing bridging and escalation in thyroid storm complicated by ALI Proposed ICU pathway for bridge therapy and escalation to TPE when thionamides are high-risk, with transition to carbimazole once hepatic function improves. Abbreviations: ALI, acute liver injury; ICU, intensive care unit; TPE, therapeutic plasma exchange Author contribution: Original figure created by B. Choudhuri (Microsoft PowerPoint); not adapted from a previously published source.

The hepatic phenotype posed a challenge for thionamide therapy, as antithyroid medications can induce liver injury. Propylthiouracil is linked with severe hepatocellular damage, while methimazole/carbimazole typically causes cholestatic or mixed injury patterns [1,12,13]. While thionamides are integral to storm management, guidelines allow pragmatic sequencing when immediate risks like hepatic failure and coagulopathy outweigh early benefits, provided alternative hormone-lowering measures are implemented [1,2,8,12,13]. The "bridge-first" approach was intended to control adrenergic and thyroid hormone effects while assessing hepatic trajectory and bleeding risk. Accordingly, the immediate goal was not to ‘avoid’ thionamides, but to buy time safely while reducing hormone action and circulating hormone burden. In settings where thionamide initiation is deferred due to marked transaminitis/coagulopathy, Figure 5 provides a practical algorithm for bridging therapy and escalation. This is not intended as a guideline but as a pragmatic workflow reflecting our case and existing therapeutic principles.

The initial strategy prioritized (i) adrenergic control, (ii) inhibition of peripheral T4-T3 conversion, and (iii) reduction of circulating thyroid hormone, while addressing critical metabolic abnormalities. Beta-blockade is essential for symptomatic control during a storm; however, its selection must be tailored to patients, particularly with bronchospasm risk and hemodynamic instability [1,2,8,9]. Corticosteroids provide hemodynamic support and reduce peripheral conversion, serving as a bridge when intrathyroidal blockade is delayed [1,2,8]. Two guideline-consistent adjuncts, cholestyramine and lithium, were used. Cholestyramine disrupts the enterohepatic circulation of thyroid hormones and expedites biochemical improvement when added to standard therapy, especially when thionamides are contraindicated [5-7,14,15]. Lithium inhibits thyroid hormone release when thionamides cannot be initiated; careful monitoring is essential due to its narrow therapeutic window in critically ill patients [14]. These agents provided a temporary biochemical "brake" until thionamide therapy could be introduced as hepatic function recovered.

During the initial phase, we did not administer iodine (SSKI/Lugol's). While iodine is recommended after thionamide administration to inhibit hormone release, giving iodine without thionamide blockade may increase hormone synthesis and worsen thyrotoxicosis. When thionamides are withheld, omitting iodine aligns with guidelines [1,2,8]. This sequencing is often overlooked in ICU settings, where treatment begins amid diagnostic uncertainty and organ dysfunction.

Another diagnostic challenge was the markedly elevated procalcitonin levels. In emergency protocols, elevated procalcitonin suggests bacterial sepsis. However, procalcitonin is a biomarker of systemic inflammatory biology rather than a definitive sepsis indicator; elevations can occur in severe non-infectious inflammatory states [16,17]. Thyroid storm may mimic septic shock with fever, tachycardia, altered sensorium, and organ dysfunction. In our patient, the lack of a localizing focus and persistently negative cultures favored thyroid storm-driven inflammation; empiric antibiotics were initiated, given early, critical illnesses, and were subsequently de-escalated in keeping with ICU antimicrobial stewardship.

Published experiences of thyroid storm with severe hepatic dysfunction highlight the heterogeneity of hepatic phenotypes and the need for individualized treatment, including extracorporeal approaches when conventional therapies fail [18-25]. Reports document cases of thyroid storm with acute liver failure, coagulopathy, and multiorgan dysfunction, requiring therapeutic plasma exchange (TPE) for stabilization or definitive therapy [18-22,24,25]. The American Society for Apheresis recognizes thyroid storm as a condition where TPE may be considered in severe cases, particularly when rapid hormone removal is needed, or standard treatments are contraindicated [18]. Evidence supports TPE as a hormone-removal strategy and transitional measure when hepatic failure limits pharmacological therapy [19-21,24,25]. In our patient, biochemical improvement and clinical stabilization through medical therapy avoided TPE escalation; however, acknowledging TPE as a contingency option aids in developing a pragmatic algorithm for similar high-risk scenarios.

In our patient, hepatic dysfunction and thrombocytopenia limited early treatment options, requiring a staged approach. Priorities included rapid adrenergic control and high-dose glucocorticoids, with cholestyramine and lithium as bridges until organ function improved for antithyroid therapy [1,2,8,14,15]. In refractory cases where standard agents remain contraindicated, TPE serves as a rescue bridge to stabilization [18-20].

This case highlights two key considerations for emergency and ICU clinicians. First, thyroid storm should be considered for "culture-negative sepsis" with atrial fibrillation, hyperthermia, and organ dysfunction, even with an unremarkable thyroid examination [1-4,8,9]. Second, management is not a rigid protocol but a sequence adapted to organ failures and contraindications while maintaining urgency [1,2,8,12-14,18].

Limitations

This is a single-case report, and causality or generalizability of the treatment sequence cannot be inferred. Our proposed algorithm is a pragmatic workflow derived from this case, and existing principles rather than a validated guideline, and management decisions should be individualized to organ failures, contraindications, and local resources.

Key learning points

Thyroid storm can closely mimic sepsis, including markedly elevated inflammatory biomarkers despite negative cultures; clinical suspicion should remain high when tachyarrhythmia coexists with biochemical thyrotoxicosis. When severe hepatic dysfunction or coagulopathy makes early thionamide therapy high risk, a temporary thionamide-sparing bridge using adrenergic blockade and corticosteroids, with adjuncts such as cholestyramine and/or lithium, can stabilize patients until liver function improves and antithyroid drugs can be introduced safely. A structured search for precipitants (infection, drug exposure, viral hepatitis, ischemia, and toxins) is essential, with early antimicrobial de-escalation when supportive evidence of infection is lacking. Cardiovascular complications may require urgent rate or rhythm control with ICU monitoring, and anticoagulation decisions should balance thromboembolic risk against bleeding risk. Definitive management of Graves’ disease should be planned after recovery (radioiodine or surgery) to reduce recurrence risk.

## Conclusions

This case shows how thyroid storm can present with a sepsis-like phenotype, malignant tachyarrhythmia, and marked hepatic dysfunction despite minimal thyroid signs, making early suspicion crucial. When severe transaminitis and coagulopathy limit thionamide use, a structured approach, early adrenergic control, and corticosteroids, with adjuncts like cholestyramine and lithium, can achieve stabilization and allow safe delayed antithyroid therapy. Careful reassessment for infectious triggers with timely antimicrobial de-escalation, along with planned Graves' management after recovery, completes a pragmatic ICU pathway for high-risk cases.
